# Construction and validation of nomogram model for predicting the risk of ventricular arrhythmia after emergency PCI in patients with acute myocardial infarction

**DOI:** 10.18632/aging.205815

**Published:** 2024-05-10

**Authors:** Wei Wang, Min Chen, Jiongchao Guo, Yuqi Wang, Jing Zhang

**Affiliations:** 1 Department of Cardiology, The Second People’s Hospital of Hefei, Hefei Hospital Affiliated to Anhui Medical University, Hefei 230000, Anhui, China; 2 Department of Cardiology, The Third Affiliated Hospital of Anhui Medical University (The First People’s Hospital of Hefei), Hefei 230000, Anhui, China

**Keywords:** AMI (acute myocardial infarction), PCI (percutaneous coronary intervention), ventricular arrhythmia, predictive model

## Abstract

Objective: To make predictions about the risk of MVA (Malignant Ventricular Arrhythmia) after primary PCI (Percutaneous Coronary Intervention) in patients with AMI (Acute Myocardial Infarction) through constructing and validating the Nomogram model.

Methods: 311 AMI patients who suffered from emergency PCI in Hefei Second People’s Hospital from January 2020 to May 2023 were selected as the training set; 253 patients suffering from the same symptom in Hefei First People’s Hospital during the same period were selected as the validation set. Risk factors were further screened by means of multivariate logistic and stepwise regression. The nomogram model was constructed, and then validated by using C-index, ROC curve, decision curve and calibration curve.

Results: Multivariate logistic analysis revealed that urea, systolic pressure, hypertension, Killip class II-IV, as well as LVEF (Left Ventricular Ejection Fraction) were all unrelated hazards for MVA after emergency PCI for AMI (P<0.05); a risk prediction nomogram model was constructed. The C-index was calculated to evaluate the predictive ability of the model. Result showed that the index of the training and the validation set was 0.783 (95% CI: 0.726-0.84) and 0.717 (95% CI: 0.65-0.784) respectively, which suggested that the model discriminated well. Meanwhile, other tools including ROC curve, calibration curve and decision curve also proved that this nomogram plays an effective role in forecasting the risk for MVA after PCI in AMI patients.

Conclusions: The study successfully built the nomogram model and made predictions for the development of MVA after PCI in AMI patients.

## INTRODUCTION

AMI (Acute myocardial infarction)’s clinical manifestation is mainly myocardial necrosis caused by acute and persistent ischemia and hypoxia of the coronary arteries [1], along with the symptom of a sudden onset, critically ill and high incidence of in-hospital mortality and cardiovascular adverse events. Despite improvements in revascularization techniques, mortality has not significantly decreased in patients with AMI [2]. In recent years, the occurrence frequency of adverse events and prognosis among elderly population has been increasing, making AMI the fatal factor of cardiac death [3]. The condition of AMI patients has the characteristics of rapid progression, rapid onset, and clinical manifestations of arrhythmia, cardiogenic shock, as well as persistent retrosternal pain. All of these were severe life-threatening factors to the patients [4]. Currently, PCI (Percutaneous coronary intervention) has become a treatment option for AMI through which the patients’ vessels can be dredged in a short time so that the blood flow can be restored. The extent of infarcted myocardium and prognosis are able to get controlled as well [5, 6]. In spite of that, complications such as coronary restenosis, ischemia, and malignant ventricular arrhythmias (MVA) after PCI [7] cannot be completely avoided. MVA is mainly characterized by severe life-threatening arrhythmias originating from the ventricles such as sustained ventricular tachycardia, ventricular flutter, and fibrillation [8]. Studies have reported that MVA occurs in nearly 5.2% of AMI cases and is a fatal factor for AMI patients [9]. MVA can lead to death due to hemodynamic abnormalities rapidly and is a serious arrhythmia in the conventional classification of the degree of arrhythmia, requiring emergency treatment [10]. Evidence suggests that malignant ventricular arrhythmias following AMI may facilitate the mortality to a 6-fold increase [11]. Guidelines recommend that patients with AMI would better live in the cardiac intensive care unit within 24- 48 hours of the onset of symptoms [12]. Therefore, early assessment of the risk of MVA in elderly AMI patients can help clinicians to take aggressive preventive and therapeutic measures, thereby reducing the risk of adverse events and improving prognosis is essential.

The nomogram prediction model can graphically display the prediction results of regression, which may help to judge the occurrence of MVA in clinical practice. Studies have shown that nomograms have a good predictive value for poor prognosis [13–15] and arrhythmia risk [16] in AMI patients. However, scanty effective tools can be applied to predicting MVA after PCI in AMI patients. The study investigated the affecting factors of MVA after PCI in AMI patients and constructed nomograms. To provide new ideas for clinical and early intervention of risk factors and improvement of prognosis.

## MATERIALS AND METHODS

### Study project

To construct the model, a total of 311 AMI patients who suffered from emergency PCI from January 2020 to May 2023 in our hospital were chosen as the training set, while 253 AMI patients who underwent emergency PCI in the First People’s Hospital of Hefei as the validation set. MVA group and non-MVA group were distinguished with each other according to whether MVA occurred after PCI in the training set. Inclusion criteria: (1) All patients met the diagnosis and treatment criteria of acute myocardial infarction [17], MVA diagnosis met the relevant diagnostic criteria [18, 19]; (2) All patients completed myocardial enzyme spectrum, troponin, coronary angiography and ECG detection, and patients can tolerate emergency PCI; (3) Aged > 18 years old; (4) Patients with integral clinical data. As for exclusion criteria: (1) previous myocardial infarction; (2) combined with severe metabolic system diseases; (3) combined with severe liver and kidney and other organ dysfunction; (4) combined with severe infection; (5) combined with coagulation dysfunction; (6) recent history of major surgical trauma; (7) malignant ventricular arrhythmia at admission before PCI. The MVAs included in this study were mainly serious life-threatening arrhythmias originating from the ventricles such as sustained ventricular tachycardia, ventricular flutter and fibrillation. The whole research received the permission of the Ethics Committee of Hefei Second People’s Hospital and the First People’s Hospital.

### Study methods

### Data collection

AMI patients’ relevant information such as demographic characteristics and clinical data were acquired from the E-medical system of the hospital, including their age, gender, BMI (Body Mass Index), hypertension, smoking status, diabetes, SBP (Systolic Blood Pressure), DBP (Diastolic Blood Pressure), Killip grade II-IV, neutrophils, lymphocytes, RBC (red blood cells), hemoglobin, platelets, albumin, urea, creatinine, uric acid, direct bilirubin, indirect bilirubin, ALT (Alanine Aminotransferase), AST (Aspartate Aminotransferase), CK (Creatine Kinase), lactate dehydrogenase (LDH), triglyceride, total cholesterol, HDL-C, LDL-C, and LVEF (Left Ventricular Ejection Fraction).

### Establishment and validation of nomogram

Stepwise regression backward method and multivariate logit regression were applied to analyzing the autonomous factors of causing MVA after PCI in AMI patients. For the purpose of predicting the risk of MVA after PCI in AMI patients, the study developed predictive nomogram models by using predictors. Besides, C-index and ROC curves were profiled to evaluate their performance. The model fit was evaluated by Hosmer-Lemeshow test, the calibration of the model was evaluated by Brier score and calibration curve was drawn; the clinical decision curve was adopted to analyze the model’s net benefit rate in the predicting process.

### Statistical approaches

The study adopted SPSS 26.0, R4.2.1 to conduct statistical analysis and plotting, using independent sample t test, enumeration data adoption rate and chi-square test to make comparisons of the measurement data between the two groups. For the purpose of figuring out the independent risk factors that affected MVA after PCI in AMI patients, the study adapts various methods including stepwise regression backward method, multivariate logistic regression, C-index, area under ROC curve, calibration curve, as well as clinical decision curve. At the same time, a collinearity test was performed and variance inflation factor (VIF) > 10 was considered collinearity. The research used two-sided tests to perform statistics. Results showed that P-value was less than 0.05, meaning statistically significant.

### Data availability statement

The data used to support the findings of this study are available from the corresponding author upon request.

## RESULTS

### Comparative study on baseline data and laboratory test indicators between the training set and validation set

Among 311 AMI projects from the training set who underwent emergency PCI, 97 developed MVA. As for the validation set, 75 of 253 developed MVA. No significant difference could be approved in gender, age, medical history, BMI, smoking status, and laboratory parameters between our two experimental groups (P > 0.05) as revealed in Table 1.

**Table 1:** Comparison of baseline data and laboratory parameters between training set and validation set in AMI patients.

**Variables**	**Training set**	**Validation set**	***t*/*χ^2^* Value**	***P-*Value**
Age (years)	61.74±13.6	59.66±14.8	1.733	0.084
Gender (Female, n%)	242 (80.1)	201 (79.4)	0.04	0.841
BMI (kg/m^2^)	24.63±3.62	24.53±3.56	0.315	0.753
Diabetes (n%)	81 (26)	52 (20.1)	3.956	0.077
Hypertension (n%)	175 (56.3)	141 (55.7)	0.016	0.898
Smoking (n%)	189 (60.8)	144 (56.9)	0.857	0.355
SBP (mmHg)	122.44±24.08	125.88±32.12	2.689	0.574
DBP (mmHg)	78.36±15.04	79.45±15.43	3.945	0.683
Killip grade II-IV (n%)	87 (28)	51 (20.2)	4.612	0.032
Neutrophils (×10^9^/L)	8.6±5.7	8.12±3.91	1.148	0.251
Lymphocytes (×10^9^/L)	1.78±1.16	1.63±0.93	1.717	0.087
RBC (×10^9^/L)	4.49±0.64	4.54±0.65	1.059	0.29
Hemoglobin (g/L)	136.68±19.66	138.51±20.83	1.068	0.286
Platelets (×10^9^/L)	205.48±63.8	215.82±67.23	1.869	0.062
Urea (mmol/L)	5.95±2.42	6.05±2.55	0.466	0.641
Creatinine (μmol/L)	86.04±34.31	87.22±61.95	2.57	0.513
Uric acid (umol/L)	361.9±102	363.73±109.23	0.206	0.837
Albumin (g/L)	40.93±3.95	40.69±3.96	5.233	0.898
ALT (μl)	62.84±87.67	55.4±115.39	0.87	0.385
AST (μl)	238.75±306.77	247.34±273.48	2.724	0.324
CK (μl)	1906.93±1804.18	1975.97±1831.45	0.449	0.654
LDH (μl)	697.15±504.41	713.38±517.3	0.376	0.707
Direct bilirubin (μmol/L)	5.35±2.62	5.55±2.86	3.455	0.708
Indirect bilirubin (μmol/L)	14.16±6.75	13.03±6.91	1.959	0.051
Triglycerides (mmol/L)	2.14±6.11	2.15±1.64	0.024	0.981
Total cholesterol (mmol/L)	4.51±1.06	4.39±1.8	2.626	0.309
LDL-C (mmol/L)	2.91±0.89	2.8±1.09	3.692	0.692
HDL-C (mmol/L)	1.1±0.25	1.1±2.59	0.02	0.984
LVEF^b^	56.69±10.41	58.02±7.72	1.688	0.092
Gensini score^b^	79.5±19.84	74.5±19.99	2.966	0.003
D-to-B time	61.23±7.88	62.5±7.22	1.986	0.047
Infarct location (n, %)			0.351	0.553
Anterior MI	159 (51.1)	123 (48.6)		
Others	152 (48.9)	130 (51.4)		
Number of diseased vessels (n, %)			0.099	0.753
1	114 (36.7)	96 (37.9)		
≥2	197 (63.3)	157 (62.1)		

BMI, Body mass index; SBP, Systolic blood pressure; DBP, Diastolic blood pressure; RBC, Red blood cell; ALT, Alanine aminotransferase; AST, Aspartate aminotransferase; CK, Creatine kinase; LDH, Lactate dehydrogenase; HDL-C, High-density lipoprotein C; LDL-C, Low-density lipoprotein C; LVEF, Left ventricular ejection fraction.

### Comparative study on baseline data and laboratory parameters of MVA after emergency PCI in AMI patients in training set

Significant differences were found in age, hypertension, SBP, DBP, Killip grade II-IV, neutrophils, hemoglobin, urea, creatinine, albumin, ALT, and LVEF levels between the group with and without MVA in the training set (P < 0.05); No significant difference could be approved in other indicators (P > 0.05), as revealed in Table 2.

**Table 2:** Comparison of baseline data and laboratory parameters between MVA and non-MVA after emergency PCI in AMI patients in training set.

**Variables**	**MVA (n=97)**	**Non-MVA (n=214)**	***t*/*χ^2^* Value**	***P-*Value**
Age (years)	65.02±14.05	60.25±13.16	2.898	0.004*
Gender (Male, n%)	70 (72.2)	172 (80.4)	2.605	0.107
BMI (kg/m^2^)	24.47±4.12	24.7±3.38	0.509	0.611
Diabetes (n%)	29 (29.9)	52 (24.3)	1.086	0.297
Hypertension (n%)	66 (68)	109 (50.9)	7.938	0.005*
Smoking (n%)	55 (56.7)	134 (62.6)	0.98	0.322
SBP (mmHg)^a^	114.9±25.95	125.86±22.42	3.798	<0.001*
DBP (mmHg)^a^	69.58±15.53	76.53±14.34	3.861	<0.001*
Killip grade II-IV (n%)	49 (50.5)	38 (17.8)	35.549	<0.001*
Neutrophils (×10^9^/L)	10.18±8.9	7.88±3.16	2.479	0.015*
Lymphocytes (×10^9^/L)	2.01±1.53	1.68±0.93	1.925	0.056
RBC (×10^9^/L)	4.38±0.64	4.53±0.64	1.917	0.056
Hemoglobin (g/L)	133.14±20.69	138.29±19.01	2.15	0.032*
Platelets (×10^9^/L)	210.95±66.81	202.99±62.39	1.019	0.309
Urea (mmol/L)	7.15±3.18	5.41±1.73	5.069	<0.001*
Creatinine (μmol/L)	88.68±53.15	70.3±18.27	3.319	0.001*
Uric acid (μmol/L)	369.28±113.3	358.55±96.55	0.86	0.391
Albumin (g/L)	37.92±4.14	39.39±3.79	3.081	0.002*
ALT (μl)	85.04±133.88	52.78±52.75	2.294	0.024*
AST (μl)	271.87±334.08	223.74±293.16	1.283	0.2
CK (μl)	2242.85±2190.49	1754.67±1581.19	1.974	0.05
LDH (μl)	774.96±617.93	661.88±440.68	1.839	0.067
Direct bilirubin (μmol/L)	5.19±2.59	5.42±2.64	0.729	0.466
Indirect bilirubin (μmol/L)	13.46±6.57	14.49±6.82	1.234	0.218
Triglycerides (mmol/L)	1.75±1.55	2.32±7.29	0.755	0.451
Total cholesterol (mmol/L)	4.36±1.06	4.58±1.06	1.653	0.099
LDL-C (mmol/L)	2.78±0.88	2.98±0.89	1.848	0.066
HDL-C (mmol/L)	1.12±0.26	1.09±0.25	1.084	0.279
LVEF^b^	53.43±10.83	58.17±9.89	3.672	<0.001*
Gensini score^b^	79.13±20.64	79.67±19.52	0.220	0.826
D-to-B time	60.2±6.41	61.69±8.44	1.554	0.121
Infarct location (n, %)			1.264	0.261
Anterior MI	45 (46.4)	114 (53.3)		
Others	52 (53.6)	100 (46.7)		
Number of diseased vessels (n, %)			0.156	0.693
1	34 (35.1)	80 (37.4)		
≥2	63 (64.9)	134 (62.6)		

BMI, Body mass index; SBP, Systolic blood pressure; DBP, Diastolic blood pressure; RBC, Red blood cell; ALT, Alanine aminotransferase; AST, Aspartate aminotransferase; CK, Creatine kinase; LDH, Lactate dehydrogenase; HDL-C, High-density lipoprotein C; LDL-C, Low-density lipoprotein C; LVEF, Left ventricular ejection fraction; *: p<0.05.

### Establishment and validation of a predictive model for the risk of MVA after PCI in AMI patients

### Risk factors analysis and feature selection of MVA after emergency PCI for AMI

**Figure 1:**
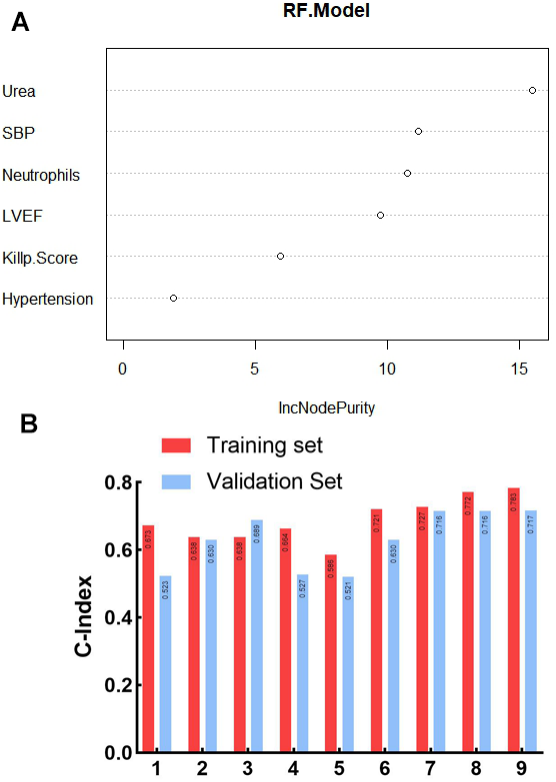
**Screening of risk factors for ventricular arrhythmia after emergency PCI in patients with AMI based on stepwise regression.** (**A**) Random forest variable importance ranking; SBP: Systolic blood pressure; LVEF: Left ventricular ejection fraction; (**B**) Consistency index of prognostic models constructed by different clinical factors on training set and validation set; 1. Urea; 2. SBP:Systolic blood pressure; 3. LVEF:Left ventricular ejection fraction; 4. Killip Score II-IV; 5. Hypertension; 6. Urea/SBP; 7. Urea/SBP/LVEF; 8. Urea/SBP/LVEF/Killip Score II-IV; 9. Urea/SBP/LVEF/Killip Score II-IV/Hypertension.

According to Table 2, several factors showed statistically significant differences (P < 0.05). Thus, they were used as independent variables, and stepwise regression was performed with whether MVA occurred after emergency PCI as the dependent variable for variable screening, and finally six important variables, hypertension, SBP, Killip grade II-IV, neutrophils, urea, and LVEF, were selected; meanwhile, the study applied random forest to evaluate the importance ranking of variables, which revealed that the factors affecting MVA after emergency PCI in AMI patients were ranked as urea, SBP, neutrophils, LVEF, Killip grade II-IV, and hypertension, as shown in Figure 1.

**Figure 2:**
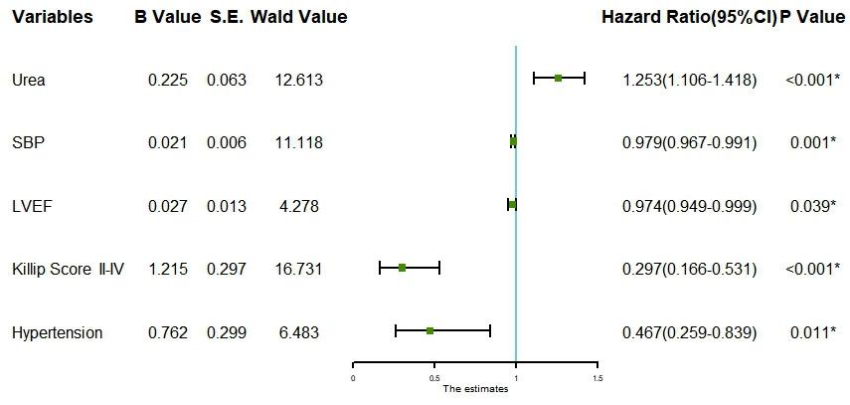
**Forest plot for multivariate logistic regression analysis.** SBP: Systolic blood pressure. LVEF: Left ventricular ejection fraction.

Taking the significant variables screened by stepwise regression as independent variables and whether MVA occurred after emergency PCI as dependent variables, multivariate Logistie regression analysis showed that hypertension, SBP, Killip grade II-IV, urea and LVEF were irrelevant risk factors for MVA after emergency PCI in AMI patients (P < 0.05), as shown in Figure 2.

### Construction of nomogram model

**Figure 3:**
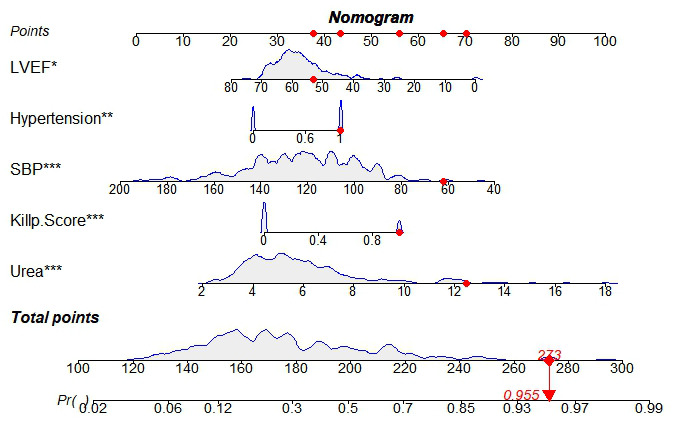
**Nomogram of ventricular arrhythmia after PCI in AMI patients.** SBP: Systolic blood pressure; LVEF: Left ventricular ejection fraction; *: P < 0.05.

For the sake of predicting the development of MVA after emergency PCI in AMI patients, the Nomogram model was drawn according to independent risk factors, the result of which was shown in Figure 3. From Figure 3, it could be sure that each predictor variable went along with a particular score on the horizontal axis of the nomogram score. Then, a total score could be calculated. It revealed from the bottom of the Nomogram a higher probability of ventricular arrhythmia’s occurrence among patients with higher total scores.

### Nomogram model validation

**Figure 4:**
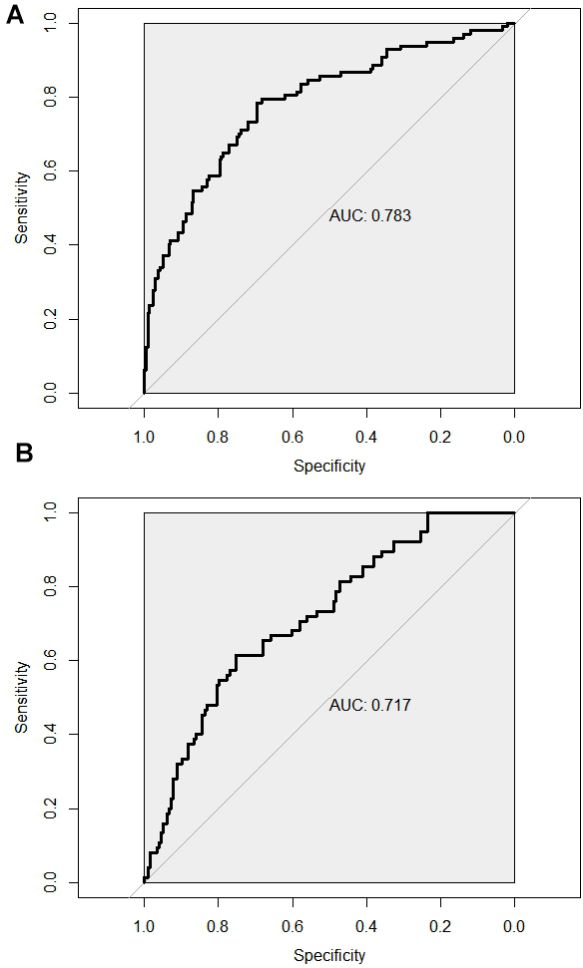
**ROC curve was used to assess the predictive efficacy of the model for predicting the risk of ventricular arrhythmia after PCI in AMI patients.** (**A**) Training set ROC curve; (**B**) Validation set ROC curve.

**Figure 5:**
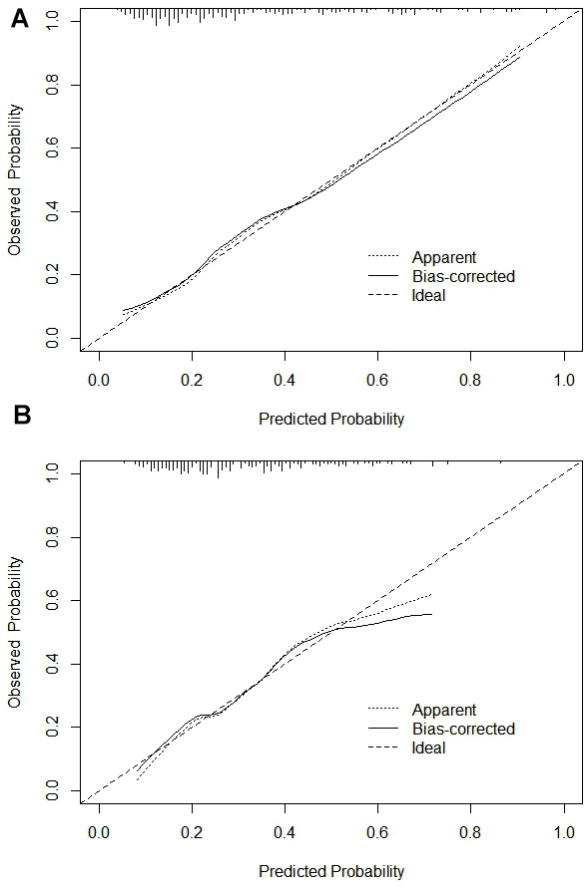
**Calibration curve.** (**A**) Training set calibration curve; (**B**) Validation set calibration curve.

The C-index of the training set and the validation set respectively were 0.783 (95% CI:0.726-0.84) and 0.717 (95% CI:0.65-0.784), which suggested that the model discriminated well. The training set ROC curve also suggests favourable model accuracy according to the result in Figure 4. Meanwhile, the results of the Hosmer-Lemeshow goodness-of-fit test showed that the model fitted well (χ^2^ = 3.4457, P=0.1786). The Brier score was not 0.164, and the calibration curve appeared to be close to the ideal curve as shown in Figure 5.

### Nomogram models’ clinical decision analysis

**Figure 6:**
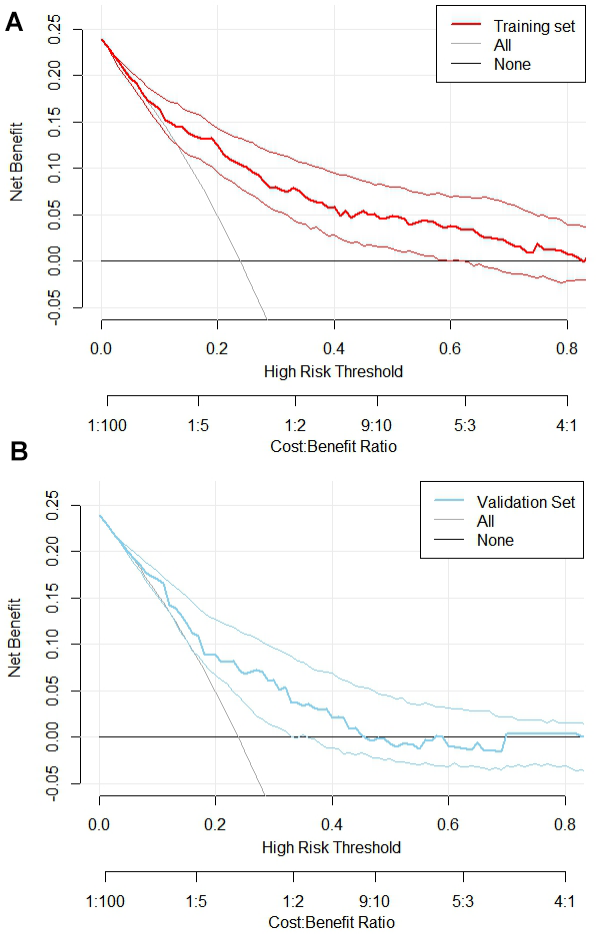
**Clinical decision curve analysis for nomogram models.** (**A**) Training Set DCA; (**B**) Validation Set DCA. The DCA was plotted with the probability of high-risk threshold as abscissa and the net benefit rate as ordinate, where the probability of high-risk threshold was set to (0, 1), the solid black line represents the net benefit rate without MVA in all patients, the solid gray line represents the net benefit rate with MVA in all patients, the red curve represents the decision curve of the nomogram model of the training set, and the blue sky represents the decision curve of the validation set

The nomogram model’s net benefit rate was higher in the range of 0.1-0.99 and 0.1-0.45 respectively in the two experimental groups, which demonstrated that it could bring net clinical benefit to patients when the threshold probabilities were 0.1-0.99 and 0.1-0.45, as shown in Figure 6.

## DISCUSSION

AMI is known as a myocardial necrosis due to the sustained disruption of myocardial blood supply, usually by atherosclerotic plaques in the coronary arteries [20]. The incidence is very high in elderly patients, and the incidence of adverse prognostic events is also very high due to the high age of patients and poor physical function. The main symptoms of AMI include severe chest pain, fatigue, dyspnea, nausea, dizziness, and sweating [21, 22]. PCI, also known as coronary angioplasty, includes rotational, directional, or atherectomy or laser angioplasty [23, 24]. Able to help the patients to rapidly restore coronary blood supply, it is widely used clinically. However, emergency patients may still suffer from the complications including coronary ischemia and ventricular arrhythmias after PCI.

As one of the most common complications of AMI, more than 80% of AMI patients have different degrees of arrhythmia. Malignant ventricular arrhythmia (MVA) is a serious complication of AMI, and 25% ~ 50% of AMI patients die of MVA [25]. MVA is an important cause of death in AMI patients [26]. MVA occurs mostly within 48 hours of AMI symptom onset, and 91% of AMI patients have been reported to develop MVA at this stage. Patients with early AMI (within 48 hours after AMI symptoms) [27] complicated by MVA have high mortality and poor prognosis, and the risk of sudden cardiac death, 30-day all-cause mortality, and 1-year mortality are higher than those of patients without MVA [28–30]. Despite the overall mortality rate of AMI patients has significantly decreased with the early diagnosis of AMI and advances in early revascularization therapy, the mortality rate of patients who develop MVA in the early stage of AMI remains high. The current study showed a higher incidence and greater harm of MVA within 48 hours (7% vs 24%) compared with the subgroup of AMI patients who developed MVA≥48 hours [31]. Most patients with AMI develop MVA outside the hospital or during emergency treatment, and AMI-induced MVA often occurs without early warning, and if MVA occurs and cannot be converted within 4-6 minutes, irreversible damage to the patient's brain tissue will occur, resulting in increased mortalit [31]. Identifying high-risk patients who develop MVA early in AMI remains therefore a critical problem.

At present, there are few predictive models for AMI complicated by MVA, and scarce effective risk prediction models are constructed for early MVA in AMI in clinical practice. Nomograms transform statistical models into visual patterns that simply and intuitively quantify risk values and assist clinicians in assessing and judging risk values for events [32].

In this study, there were 311 training sets, among which 97 AMI patients developed MVA after PCI, with an incidence of 31.19%. In the validation set of 253 patients, 75 AMI patients developed MVA after PCI, with an incidence of 29.64%. In this study, the results of stepwise regression screening and multivariate logistic regression analysis suggested that hypertension, SBP, Killip grade II-IV, urea, and LVEF were all independent risk factors for ventricular arrhythmia after emergency PCI for AMI.

This study found that systolic blood pressure was lower and Killip class was higher in AMI patients who developed MVA after PCI than in those who did not develop MVA. It predicts the risk of early MVA in AMI patients, which is consistent with previous findings that hypotension and Killip class > II are risk factors for MAV in AMI, and recurrent MVA in AMI patients that is, the presence of “electrical storm” is significantly associated with Killip class ≥ II and more extensive myocardial infarction (elevated CK peaks) at admission [33]. Severely impaired LVEF (< 30-40%) indicates worse left ventricular systolic function, lower systolic blood pressure as well as larger myocardial infarct size after AMI. Thus, it has been reported to be strongly associated with adverse events and high mortality after AMI [34]. This study suggests that AMI patients with lower left ventricular ejection fraction are more prone to fatal ventricular arrhythmias during hospitalization. Similar conclusions were recently reported by Albanese et al. [35]. It is worth mentioning that concurrent fatal arrhythmia in AMI patients in our study was also significantly associated with severe hemodynamic impairment (Killip class II-IV), consistent with previous findings [33]. We found that there may be a large number of patients with so-called secondary (non-primary) ventricular fibrillation in this population. Secondary ventricular fibrillation is commonly defined as ventricular fibrillation occurring in the setting of severe heart failure and cardiogenic shock, and has been shown to have a very high in-hospital mortality (up to approximately 50% at 1 month) compared with control subjects [36].

Previous studies have demonstrated the fact that AMI patients’ renal dysfunction plays an essential role in predicting hospitalization rate and long-term mortality [37]. In a similar way, serum creatinine levels are bound up with prognosis [38]. The research results have showed that creatinine and urea levels were obviously higher in the group with MVA than that without MVA (P<0.05). Elevated blood urea levels were independent risk factors for MVA after PCI in AMI patients. Therefore, in order to achieve an ultimately effective reduction of mortality and improve prognosis, it is necessary to use serum creatinine and urea levels as crucial predictors of prognosis during the formulation process of individual-based treatment. Moreover, risk stratification should be conducted according to renal function status and blood urea nitrogen and creatinine levels.

It has been demonstrated that hypertension is a high-risk cause of coronary heart disease [39]. Myocardial infarction is one of the most common cardiac insults in hypertensive patients. Hypertensive patients have left ventricular hypertrophy due to increased long-term peripheral resistance. Left ventricular hypertrophy predisposes to myocardial infarction due to increased oxygen consumption and relatively reduced myocardial capillary density. Target organs, especially the heart, are compromised in hypertensive patients. Chances are that MVA is caused by myocardial ischemia, reduced cardiac systolic as well as diastolic function.

For the sake of effectively predicting the risk of MVA after PCI in AMI patients, a nomogram model was developed and validated to perform risk prediction according to indicators with statistical differences after stepwise regression screening and multivariate logistic regression analysis. It seems that the nomogram model predicted the C-index of MVA after PCI in AMI patients better. The accuracy of the nomogram model was further demonstrated by calibration and DCA analysis.

The flaws of this study are listed as follows: firstly, it is two-centered with limited sample size and probably not objective enough; also, the model validation still needs to be improved for its lack of study center, undersized sample as well as lacking of perspectiveness. In conclusion, to improve the clinical applicability of this model to help further exploring the risk factors of MVA after PCI in AMI patients and optimizing the nomogram model, large-scale project, multi-center clinical data are still indispensable.

## CONCLUSIONS

To summarize, urea, systolic blood pressure, hypertension, Killip class II-IV as well as LVEF were risk factors for MVA risk after PCI in AMI patients. The nomogram model to predict the risk of MVA after PCI in AMI patients developed in this study based on the above risk factors is of good discrimination, calibration and clinical effectiveness, and is proved to be resultful in making risk predictions in early clinical period of MVA after PCI in AMI patients. Also, it is of great value in promoting the medical achievements in the areas of early detection, early reporting and early treatment, counting a lot for the prognosis.
